# Spatially resolved photoresponse on individual ZnO nanorods: correlating morphology, defects and conductivity

**DOI:** 10.1038/srep28468

**Published:** 2016-06-23

**Authors:** K. Bandopadhyay, J. Mitra

**Affiliations:** 1 School of Physics, Indian Institute of Science Education and Research, Thiruvananthapuram, 695016, India

## Abstract

Electrically active native point defects have a significant impact on the optical and electrical properties of ZnO nanostructures. Control of defect distribution and a detailed understanding of their physical properties are central to designing ZnO in novel functional forms and architecture, which ultimately decides device performance. Defect control is primarily achieved by either engineering nanostructure morphology by tailoring growth techniques or doping. Here, we report conducting atomic force microscopy studies of spatially resolved photoresponse properties on ZnO nanorod surfaces. The photoresponse for super-band gap, ultraviolet excitations show a direct correlation between surface morphology and photoactivity localization. Additionally, the system exhibits significant photoresponse with sub-bandgap, green illumination; the signature energy associated with the deep level oxygen vacancy states. While the local current-voltage characteristics provide evidence of multiple transport processes and quantifies the photoresponse, the local time-resolved photoresponse data evidences large variations in response times (90 ms–50 s), across the surface of a nanorod. The spatially varied photoconductance and the range in temporal response display a complex interplay of morphology, defects and connectivity that brings about the true colour of these ZnO nanostructures.

There have been several exciting advances in the multifunctional applications of ZnO nanostructures^1^,^2^,^3^, from piezoelectric nanogenerators^4^ to gas-sensors^5^ and light emitting diodes^6^ to photodetectors^7^. Many of the above stem from the non-centrosymmetry of its wurtzite structure and the direct band gap (3.3 eV) coupled with a large exciton binding energy^8^ (~60 meV). The *n*-type electrical conductivity of *undoped* ZnO has been understood to originate from an interplay of its non-stoichiometric native point defects (NPDs)^9^,^10^ i.e. Zn interstitials (I_Zn_) and O vacancies (V_O_). In nanostructured ZnO, tunability of the NPDs and their dynamic redistribution capability, especially at surfaces (and interfaces) has made the system more *colourful* for exploitation. The large surface to volume ratio of the nanostructures, which has been key to many applications, has also been seen to strongly influence the NPDs. The abundance of these NPDs has been successfully controlled by engineering the morphology of the nanostructures, through tailoring the growth techniques^11^, conditions^12^ and doping. Understanding this correlation of morphology with electrical transport has become central to optimising the ensuing device properties. The presence of NPDs in these nanostructures is directly evidenced in the luminescence properties^8^,^13^. A typical photoluminescence spectrum depicts the characteristic near-band-edge emission in the UV, along with several prominent emissions in the violet, green, orange and red, indicating the presence of shallow and deep level defect states^10^,^14^,^15^. The broad green emissions have been extensively reported^15^,^16^,^17^,^18^, which likely originate from *electron-hole* recombination between a singly ionised oxygen vacancy state (V_O_^+^) and the valence band of ZnO. However, fewer studies have reported a corresponding electrical photoresponse of ZnO with green^19^,^20^ light, unlike more prevalent super- band gap excitations^12^.

Here, we report conductive atomic force microscopy (CAFM) studies of spatially resolved photoresponse on individual, vertically oriented ZnO nanorods (ZNR). Though morphological and structural characterization of such samples is more routinely undertaken by transmission electron microscopy (TEM)^21^ and scanning electron microscopy (SEM)^8^ and its associated techniques e.g. cathodoluminescence (CL)^13^,^22^, CAFM provides the capability to map topography simultaneously with high-resolution electrical characterization^23^,^24^, not afforded by the other techniques. When coupled with light injection capability into the AFM junction it then enables us to record spatially resolved photocurrents^25^,^26^, with nanometer resolution and their correlation to surface morphology. In operation, the conducting CAFM tip forms a nanoscale Schottky junction with the *n*-type ZnO allowing local current–voltage (*IV*) measurements, which has been used to obtain current maps in the dark^27^. Point *IV* measurements with and without illumination^28^ quantifies the photoresponse, which may be used to study mechanisms of charge transport on specific photoactive nanostructures^29^,^30^. Though the contrast in the current maps may be used to infer surface conductivity variation, it cannot quantify the conductance change. Here, we have investigated the variation in local conductivity and photoconductivity by directly recording the local d*I*/d*V* or conductance maps (CMAPs)^31^ on ZNRs, to visualise and quantify the spatial variation in surface conductance, with and without optical excitation. The CMAPs offer an alternative in probing spatially resolved photoresponse and on occasion a more sensitive probe in evidencing NPD distribution^31^, in comparison to the total current map. The CMAPs presented here not only demonstrate a direct correlation between surface morphology (grain structure) and local conductivity but surprisingly also with the local electrical photoresponse. Finally, local variations in transient photoresponse are studied to demonstrate the inherent non-uniformity in photoactivity on these nanorods. Overall, the present investigation provides critical inputs to design better and faster ZnO nanostructures for optoelectronic applications.

## Experimental Details

**Figure 1:**
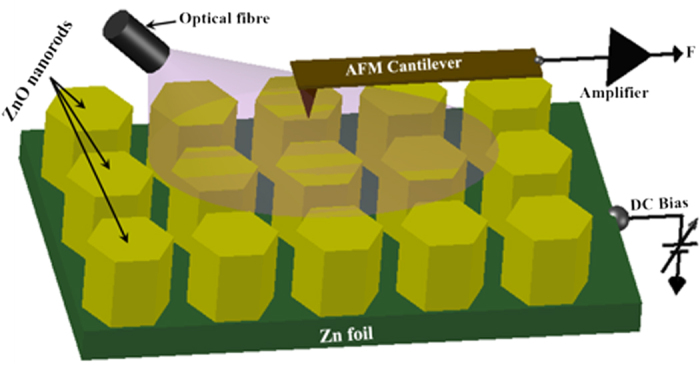
Schematic of the atomic force microscope setup, modified with the optical fibre insert for illuminating the junction.

Vertically aligned ZnO nanorods (ZNRs) were grown on Zn foil by an oxidation method that results in nanorods with an abundance of NPDs i.e. Zn interstitials and oxygen vacancies, ensuring higher conductivity, as reported in a previous study^10^. Detailed synthesis and structural characterization data (TEM images are shown in Figure S1 in Supplementary Information) have been reported elsewhere^10^. The structural data shows these hexagonal ZNRs to be dominantly (0002) oriented with six crystallographic equivalent (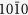) side planes, with an average diameter ~400 nm and ~4 μm in height. The CAFM studies have been conducted using a Bruker Multi Mode 8™ AFM equipped with an extended TUNA™ module to record the tip-sample current in contact mode, within a range of 1.2 fA to 1.2 μA. While the tapping mode topography images were recorded with BRUKER RTESP cantilevers (Sb doped Si, *f* = 339–352 kHz), the CAFM images were acquired using MikroMasch gold coated probes (HQ:CSC37/Cr-Au, *k* = 0.1–0.6 N/m). The time-resolved photocurrent measurements were performed using stiffer doped diamond coated probes (Veeco DDESP-10, *k* = 20–80 N/m), for improved junction stability. Current vs. voltage (*IV*) characteristics, were recorded using both the conductive probes. CMAPs are 2D plots of the local *dI/dV* of a surface, recorded simultaneously with topography and current map in the contact mode. For recording the CMAPs, the dc sample bias (*V*_DC_) was modulated with an ac bias (*V*_AC_), such that (*V*_AC_)_*rms*_ ≤ 5% of *V*_DC_. For *V*_DC_ = −2 V, *V*_AC_ = 100 mV (p-p) has been used. A digital signal generator was used to source the *V*_AC_ and the *V*_rms_ equivalent of the in-phase current signal was detected using a digital lock-in amplifier. This *V*_rms_ response is proportional to the local d*I/*d*V*|*V*_DC_ and is plotted to generate the CMAPs. Further details on the CMAP technique are available in the Supplementary Information. Voltages noted in all cases were applied to the sample, with the tip held at virtual ground. While performing the photoresponse measurements, the tip-sample junction was illuminated sequentially with two wavelengths of light from continuous wave lasers, through a coupled 800 μm core optical fibre held at a distance of ~1 mm from the junction, at an angle of 45° (schematically shown in Fig. 1). The laser power at the sample surface for λ_1_ (355 nm ± 3 nm) and λ_2_ (532 nm ± 3 nm) was 6 mW and 9 mW, respectively. While λ_1_ corresponds to a super- bandgap energy, *h*ν > 3.3 eV, the second wavelength λ_2_ with *h*ν~2.33 eV was specifically chosen to lie within the green emission band observed from these nanorods^10^. It corresponds to the energy difference between the singly ionised V_O_^+^ states and the valence band of ZnO^10^,^18^. All samples were stored in the dark for an extended period between experiments and all AFM experiments were conducted in ambient, in dark or with illumination, as specified.

## Results and Discussion

**Figure 2:**
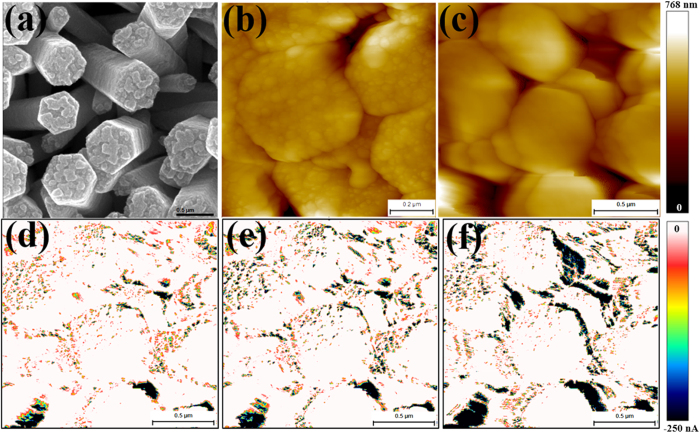
(**a**) SEM image of hexagonal ZnO nanorods (**b**) tapping mode AFM topography of nanorod surface; (**c**) CAFM topography and the simultaneously acquired current maps at sample bias of (**d**) −1.0 V (**e**) −1.5 V (**f**) −2.0 V. (current range in (**d–f**) are in 0 to −250 nA).

**Figure 3:**
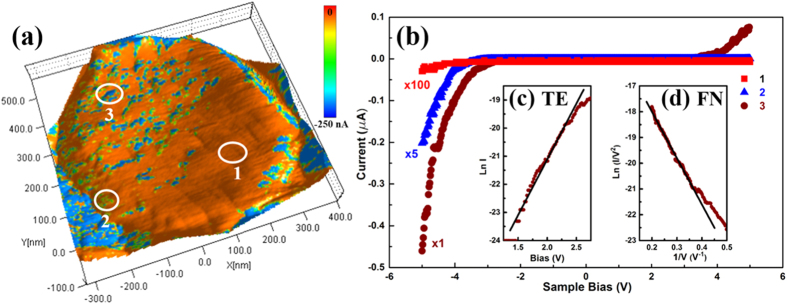
(**a**) Overlaid current map (at −2 V) on the 3D topography image of a ZnO nanorod. White circles demarcate areas of (1) low (2) medium and (3) high current. (**b**) Point *IV* characteristics recorded within the three current regions, the current values for (1) and (2) have been scaled by factors of 100 and 5 respectively, for clarity. Insets: (**c**) thermionic emission and (**d**) Fowler-Nordheim fits (see text) to forward bias *IV* data obtained at region 3.

Figures 2a,b show the SEM image and AFM topography acquired in tapping mode on the same sample. Owing to the exceptionally high depth of field afforded by the SEM, the image in Fig. (2a) distinctly resolves the top surface as well as the side planes of the ZNRs. A majority of the side planes are not evidenced to be of a single crystallographic origin but are suggestive of ZNR formation by a ‘stack’ of hexagonal (002) oriented crystallites. Similarly, the top hexagonal facets of the ZNRs are seldom atomically flat, but exhibit a broad grain shape and size distribution. Measured over multiple ZNRs, the grain size varies between 20–200 nm with an average grain size ~30 nm (Figure S2 in Supplementary Information). Interestingly, both disorder of the side planes and granularity of the top facets are highly correlated with the local conductivity of the ZNRs. Figure 2(c) shows the contact mode topography of a cluster of ZNRs, along with the CAFM current maps (Fig. 2d–f) acquired at *V*_DC_ of −1V, −1.5V and −2V, in the dark under forward biased (FB) configuration. The current maps (Fig. 2d–f) show a current variation of 0–250 nA with the darker shades denoting the higher currents. Evidently, the high current regions in the current maps are located at the edges and the side planes of the nanorods^27^. The bias dependence not only elucidates the relative abundance of the high current regions but also demonstrates that its correlation with the side planes is not merely topographical. That is, not all side planes seen in the topography image correspond to dark regions in the current maps, affirming that the current spikes are not due to sudden changes in the feedback or loading force at edges, where there are significant height variations. A recent density functional investigation^32^ shows that upward band bending at the non-polar (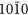) side planes of the ZNRs lowers the surface band gap (E_g_) by 0.31 eV from the bulk value. Such a large decrease in surface E_g_ will directly impact the population of electrons in the conduction band (CB) due to the exponential interdependence of the CB electron density with E_g_, ensuring a large gain (~10^2^) in number density on the side planes. The side planes of similar ZNRs have also been shown to contain a high incidence of oxygen vacancies, reflected in an enhanced green emission from the side planes, in spatially resolved CL investigations^13^. The current on the side planes would benefit from both the above, lower E_g_ and a higher density of NPDs. The high current regions on the ZNR top surface appear highly dispersed with non-uniform coverage (Figures 2 and 3 and S3). An overlay of the current maps recorded at −2.0 V, on the corresponding topography (Fig. 3a) shows that the current spikes appear only at regions with large surface roughness i.e. areas with smaller grains, harbouring higher density of NPDs^17^. This co-localization of granularity, disorder and high current is rather counterintuitive since high current, representative of high carrier density and local conductivity, does not correlate positively with disorder in general. But, in the context of ZnO, a system in which the electrical (*n*-type) conductivity itself stems from NPD induced doping^10^, this is perhaps not entirely surprising. Disorder though does hamper the local conductivity, as we shall see later.

In order to obtain more detailed information regarding the local electrical properties, *IV* characteristics were recorded between ±5 V, at multiple points on the hexagonal top surface of individual ZNRs. Figure 3b shows a set of dark *IV*s recorded at points located within three regions, demarcated by circles in the overlaid current map shown in Fig. 3a. The circles correspond to regions of (1) low current, (2) medium current and (3) high current. *IV* data in all three regions show rectifying behaviour with a high rectification ratio (>10^2^), characteristic of Schottky junctions, formed between the Au tip and *n*-type ZnO. Current values from regions 2 and 1 (Fig. 3b) have been scaled by factors of ×5 and ×100 for clarity. It is worth noting that even in the FB regime, at −5 V, the average current in the “high current” region is 10^3^ times higher than that in the “low current” region. The rectifying nature of the *IV* data and the high applied biases that are comparable to the work function (5.1 eV) of Au and the electron affinity (4.2 eV) of ZnO indicate that electrical transport across the tip-sample junction is associated with multiple transport processes ranging from thermionic^29^ to field emission^33^. Consequently, the FB *IV* characteristics were analysed using (i) the thermionic emission (TE) theory, for low biases (|V| < 2 V) and (ii) field emission theory, for |V| > 2 V. Following the standard TE model^34^ analysis, Fig. 3c shows a linear fit to ln(*I*) vs. *V* plot used to estimate the barrier height *φ*_*B*_ and ideality factor *ɳ*. The Richardson constant of 32 A cm^−2 ^K^−2^ (*m*_*eff*_ = 0.27 *m*_*e*_ for ZnO^34^) was used along with an estimated contact area of 16.6 nm^2^, calculated^35^ for a tip loading force of 27 nN. The average values calculated for *φ*_*B*_ and *ɳ* were 370 ± 30 meV and 8.5 ± 0.5, respectively. While the value for *φ*_*B*_ is comparable to existing reports^36^, the higher value of *ɳ* is indicative of the non-thermionic contributions to electrical transport across the barrier. The non-thermionic contribution is also highlighted via the departure from linearity seen for |*V|* > 2.0 V, above which field assisted tunnelling becomes significant. Figure 3d shows a linear inter-dependence between ln(*I/V*^2^) and 1/*V*, for |*V*| > 2.5 V, representative of the Fowler-Nordheim (FN) tunnelling mechanism. The slope of the linear region yields a barrier height of 500 ± 150 meV. The large uncertainty primarily arises from the large uncertainty in estimating the barrier width^17^,^37^. Again, the calculated *φ*_*B*_ resides broadly within the range of previous reports, 350–800 meV, for various Au-ZnO Schottky junctions^36^.

**Figure 4:**
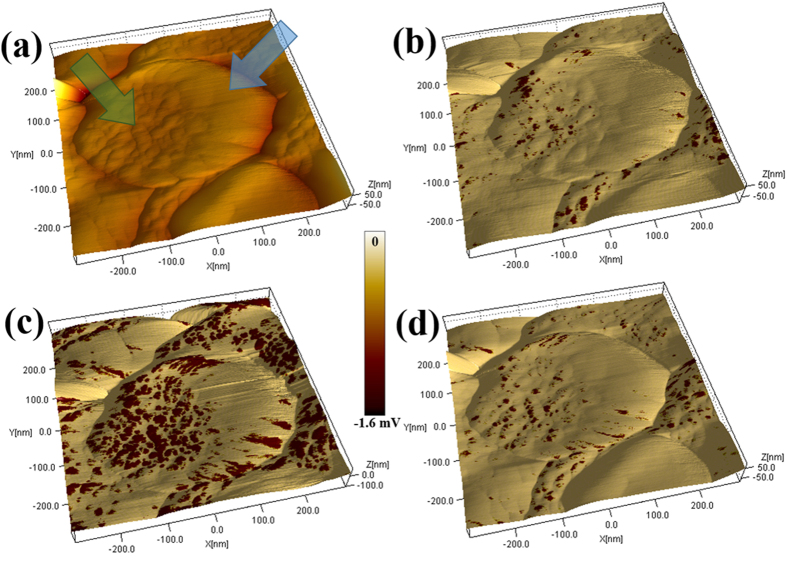
(**a**) 3D topography image of a ZnO nanorod; the green and blue arrows indicate regions with smaller and larger grain structure. Corresponding *dI/dV* maps at (**b**) dark and upon (**c**) *λ*_*1*_ (355 nm) and (**d**) *λ*_*2*_ (532 nm) illumination.

**Figure 5:**
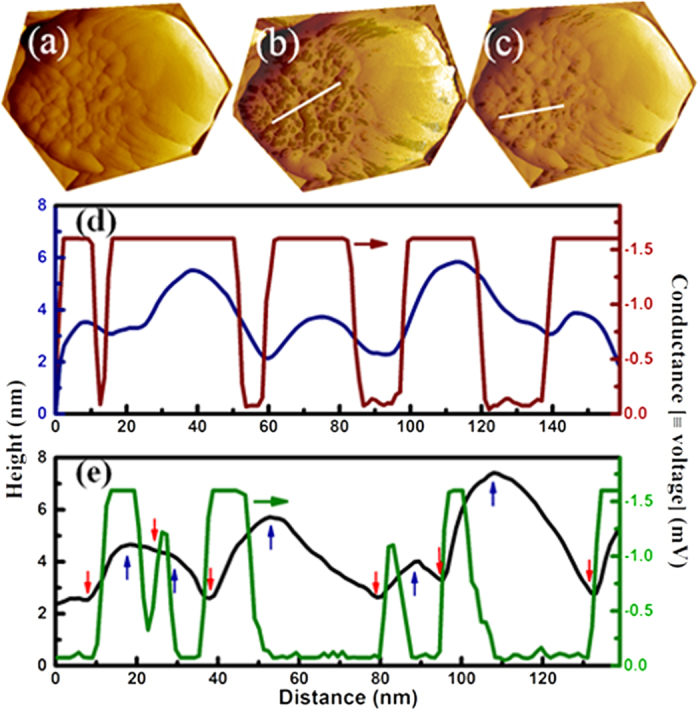
(**a**) Topography of ZnO nanorod, (**b**,**c**) topography overlaid with CMAP for 355 and 532 nm excitation, (**d**,**e**) topography and CMAP line scans taken along white lines in (**b**,**c**), respectively. The blue and red arrows in (**e**) denote the grain centres and boundaries respectively.

Figure 4a shows the 3D topography of a ZNR top surface, indicating the presence of small grains (diameter ~30 nm) clustered towards the left (green arrow) and larger elliptical grains (longest dimension ~150 nm) to the right (blue arrow). A ZNR with such distinct variation in grain size was specifically chosen to accentuate the grain size and disorder effect on local conductance and electrical photoresponse. Figures 4b–d show the topography overlaid with *dI/dV* maps (CMAPs), acquired with −2 V bias in dark and with *λ*_*1*_ (355 nm) and *λ*_*2*_ (532 nm) illumination respectively. With the darker shades denoting higher conductance, the CMAP acquired in the dark (Fig. 4b) shows relatively few of the smaller grains with high conductance, while the larger grains show little or no conductance, as seen in the current maps. Illuminating the junction with *λ*_*1*_ (Fig. 4c) results in a substantial increase in conductance on most of the smaller grains, indicating photogeneration of a large number of carriers, excited across the band gap. Notably, a conductance enhancement is also observed for the junction illuminated with *λ*_*2*_, the sub-band gap excitation (Fig. 4d), with the enhancement localised primarily in the region with the smaller grains. The contrast observed in the CMAPs, at −2 V sample bias, essentially provides a map of the surface electron density and charge transfer probability, analogous to their interpretation in the context of scanning tunnelling spectroscopy^31^. Further, since the change in local conductance here is related to the photo-generation of charge carriers, the CMAPs directly reflect on the spatial distribution of photoactivity. The overlays depict two very interesting facts; firstly, the smaller grains are significantly more photoactive for super- band gap excitation (*λ*_1_) compared to the larger grains, analogous to the observed difference in the current maps. The larger grains display minimal conductivity and photoactivity. This inhomogeneity in photoresponse is surprising if the conductivity enhancement is only decided by *e-h* excitation across the band gap. Secondly, the overlays also show that the high photoconductivity is dominantly localised at the centre of the individual photoactive grains, under *λ*_*1*_ illumination and is lower at the grain edges (see Figure S4 and movie M1 in the Supplementary Information). Under green (*λ*_*2*_) excitation, the high photoconductive regions are more correlated with the grain boundaries than the grain centres. The correlation is further evident in the synchronised line scans taken across topography and the corresponding CMAPs shown in Fig. 5 and Figure S6 in the Supplementary Information. Grain boundaries, being structurally disordered and harbouring a higher density of defects are generically associated with low electrical conductivity. The second observation assumes significance in view of the suggested localization of NPDs, specifically the oxygen vacancies (*V*_*O*_) at the grain boundaries and surfaces. Crucially, their abundance is strongly affected under photo-excitation and is reflected in various physical properties, e.g. electrical conductivity^20^, luminescence^10^,^38^ and paramagnetic resonance^17^. Its worth noting that the above spatial variation in conductance and photoconductance and their correlation with grain size are observed abundantly across all the photoactive ZNRs we have studied, as seen in the additional topography and CMAP data presented in Figures S5 and S6 in the Supplementary Information. Figure S5 shows the CMAP in dark and with *λ*_*1*_ excitation on a cluster of ZNRs of different sizes varying from 200–500 nm, reinforcing the correlation with grain size. The widths of the ZNRs are seen to play a secondary role in deciding the photoresponse, which definitely benefits from the geometry of the nanorods i.e. the large surface to volume ratio.

**Figure 6:**
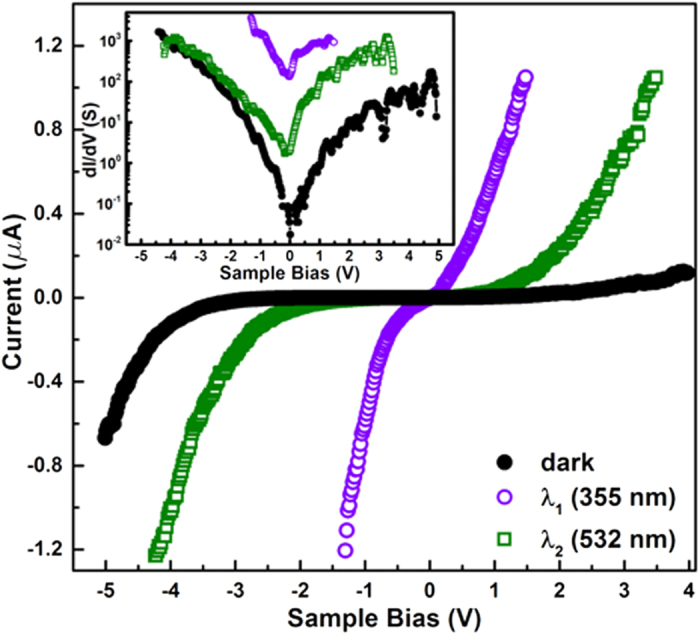
Point *IV* spectra in dark and with λ_1_ (355 nm) and λ_2_ (532 nm) illuminations. Inset shows the variation of junction conductance (*dI/dV*) with bias for the *IV*’s displayed.

To quantify the photoactivity of the *active* grains point *IV* characteristics were recorded, with doped diamond coated tips, with and without illumination, as shown in Fig. 6. *λ*_1_ illumination induces a photocurrent over 10^4^ times its dark value, beyond 1V RB. The inset shows the corresponding d*I/*d*V vs.* bias variation, showing a junction conductivity enhancement ~4 orders of magnitude at zero bias. The sub-bandgap, *λ*_*2*_ illumination shows much enhanced photoresponse under RB than FB, with a maximum current increase ~750 times in reverse compared to an increase ~5 under FB. This intriguing observation is consistent with the photoresponse of planar ZnO-PEDOT:PSS *pn* junction diodes reported earlier^20^. As mentioned before, the intrinsic *n*-type conductivity of the ZNRs likely stems from an interplay of two NPDs, V_O_ and I_Zn_ that donate electrons to the CB^10^. Our results, recorded in the dark, indicate that the smaller grains harbour relatively larger fractions of these defects compared to the larger grains, resulting in their higher local conductivity (Figs 3 and 4, S3 and S4). However, the local conductivity is adversely affected by band bending at the grain surfaces^17^,^38^,^39^ and boundaries^37^, through an increase in the built-in potential and the depletion width, at the junction. Additionally, band bending also controls the relative abundance of the V_O_ and their oxidation states (V_O_^+^/V_O_^++^)^40^ which indirectly affect the free electron density in the system. The larger availability of free electrons in the smaller grains then facilitates adsorption of atmospheric oxygen^41^ selectively onto themselves, thereby reducing the local carrier density. Super- bandgap (λ_1_) excitation would directly increase the CB electron density due to photoexcitation. Additionally, the photogenerated holes migrating to the interface (junction), especially in the RB, neutralise the adsorbed oxygen, which desorbs releasing the trapped electrons. These *freed* electrons along with their photogenerated counterparts then contribute to the exponential increase in local conductivity. The observed conductivity enhancement is also aided by the simultaneous lowering of the barrier height^11^ at the desorbed oxygen sites, on the smaller grains (Fig. 4). The highly disordered grain boundaries being resistive to electrical transport naturally limits the photoresponse away from the boundaries, limiting the photoresponse to the grain centres (Figs 5 and S5 and S6). Under λ_2_ illumination, electrons are excited from the VB to the intermediate V_O_^+^ states^20^, leaving behind holes in the VB. The excited electrons, confined to the narrow defect band contribute little to the overall conductivity. Therefore, the conductivity increase is primarily due to the photogenerated holes and their interaction with the adsorbed oxygen^19^, as in the case of λ_1_ excitation. It is worth noting that bare ZnO does not show photoresponse to green illumination^20^, but does so under biased *p-n*, Schottky or tunnel junction conditions, where interfacial or local band-bending is understood to play a significant role in deciding the oxidation state of the NPDs. Previous tunnelling experiments on ZnO indicate that the tunnel current benefits from the direct involvement of donor states in the charge transfer process^31^. Thus, simultaneous optical excitation coupling onto the dopant states would readily affect the junction current and conductance as seen above. The effect is accentuated in the RB regime since it facilitates transport of holes to the surface i.e. at the junction and is greatly subdued under FB conditions, which also inhibits ionisation of the local donor states due to reduced band bending at the junction. Lastly, the observed localization of high photoresponse close to the grain boundaries, selectively for λ_2_ excitation provides evidence of a higher density of oxygen vacancies closer to the grain boundaries, which though expected had not been directly mapped before.

**Figure 7:**
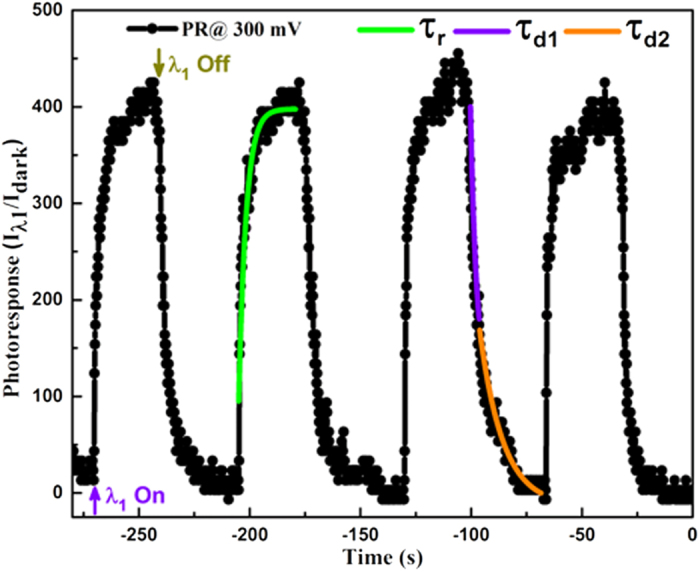
Transient photoresponse of a single grain upon λ_1_ (355 nm) excitation at a sample bias of +300 mV.

**Figure 8:**
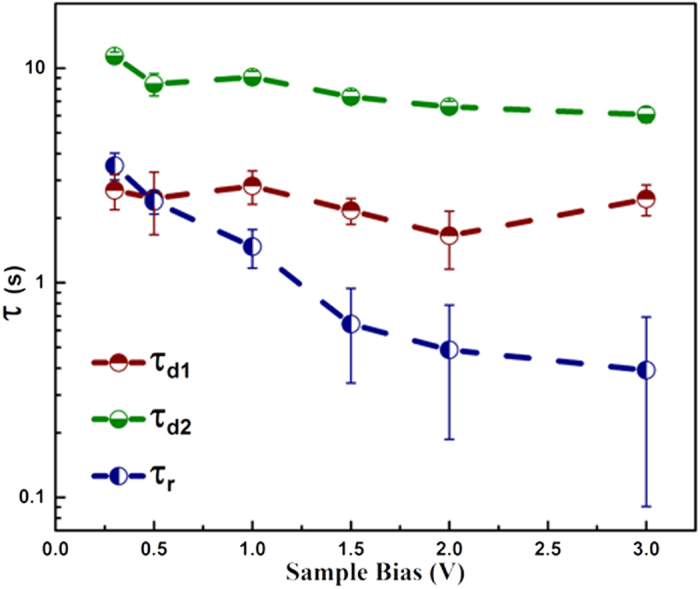
Variation of the photoresponse rise and decay time constants with applied bias. The error bars indicate the range of values obtained from photoresponse of various active grains.

The spatial variation in the electrical conductivity on the top surface is also accompanied by a local variation in the transient photoresponse between the *active* and *passive* grains. Figure 7 shows the time resolved photoresponse of the junction at a point on one such photoactive grain, at +300 mV RB under λ_1_ illumination. The switching current curves show an on/off ratio of ≈400 and an exponential time dependence of the rising and decaying photocurrent. The rise and decay time constants were obtained by fits (shown in Fig. 7) of the relevant sections of the transient photoresponse to the following equations;


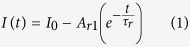



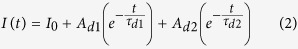


where *I*_*0*_ is the dark current, *A*_*r1*_*, A*_*d1*_*, A*_*d2*_ are positive constants. τ_*r*_ and τ_*d1*_, τ_*d2*_ are the time constants for rising and decaying photocurrent, respectively. Figure 8 shows the bias dependence of the various time constants, calculated from the transient photoresponse of multiple photoactive grains. The error bars, in Fig. 8 denote the range of time constants estimated at each bias value. Average τ_*r*_ decreases from 3s to 400 ms between 0.25–3 V; with a lowest detected value of 90 ms. The generic lowering of the junction time-constant with increasing RB, is primarily due to the increasing depletion width, which progressively decreases the junction capacitance (*C*_*D*_). For a planar Schottky junction in the RB, *C*_*D*_ is given by^34^;


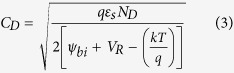


where *ε*_*s*_ is the dielectric permittivity of the semiconductor, *ψ*_*b*i_ is the built-in potential, *V*_*R*_ the RB potential, *k* is the Boltzmann constant, *N*_*D*_ the carrier density and *T* the temperature. An increasing *V*_*R*_ would decrease *C*_*D*_, in turn decreasing the time-constant. The decay section of the curves is characterised by two distinct time-constants τ_*d1*_ and τ_*d2*_ relevant at different time scales. The faster, *τ*_d1_ has an average value of 2.5 ± 0.3 s without any systematic bias dependence. However, the slower *τ*_d2_ decreases from 10 s to 7 s with increasing bias. The presence of two decay time constants indicates the existence of two parallel channels that control the carrier density in the ZNRs. The fast quenching process τ_d1_, is likely due to the recombination of the photogenerated carriers^42^, around the junction. With the UV excitation turned off the free electron cloud is partially used to locally re-adsorb O_2_ molecules onto the ZNR surface. This is expected to be a slower process, compared to *e-h* recombination, thus decreasing the free electron density on a longer timescale, τ_d2_. Its bias dependence likely arises from the variation in the depletion width. It is worth reiterating that the signals detected here are only responsive to the optoelectronics of the junction and its immediate surroundings, due to the significantly smaller contact area of tip-sample junction than its planer counterparts, which also results in the fast response times observed above.

## Conclusion

In conclusion, this study reveals significant spatial variation in electrical conductivity and photoresponse, on individual nanorods of ZnO, which are strongly correlated with grain size distribution on the hexagonal top surface of the nanorods. Differences in spatially resolved conductance maps with super- (UV) and sub- (green) bandgap excitations then discriminate between the photoconductivity originating solely from photoexcitation of charge carriers across the band gap and atmospheric oxygen mediated carrier generation. The conductance maps also provide direct evidence of increased localization of the defects states close to the disordered grain boundaries. Thermionic emission and tunnelling models were used to comprehend the bias dependent charge transfer observed across the AFM tip–ZnO nanorod junction. Fits to the *IV* characteristics were then used to calculate the barrier properties; the junctions were found to be non-ideal with barrier heights ranging between 0.3–0.5 eV. Our results demonstrate that a smaller grain size (<50 nm) distribution enhances electrical conductivity and photoresponse in *undoped* ZnO nanostructures. The photoactive smaller grains are also significantly faster in switching between the low and high conductivity states with response times below 100 ms. This investigation demonstrates that spatially resolved conductance maps provide an alternate technique for probing the local photoresponse on nanostructured systems and the results provide critical inputs to engineering ZnO nanostructures with higher photoresponse and smaller response time.

## Additional Information

**How to cite this article**: Bandopadhyay, K. and Mitra, J. Spatially resolved photoresponse on individual ZnO nanorods: correlating morphology, defects and conductivity. *Sci. Rep.* **6**, 28468; doi: 10.1038/srep28468 (2016).

## Supplementary Material

Supplementary Information

Supplementary Movie M1
