# Supplementary material for: Spatially resolved photoresponse on individual ZnO nanorods: correlating morphology, defects and conductivity
Source: Sci Rep. 2016 Jun 23;6:28468. doi: 10.1038/srep28468 (PMC4917851; doi:10.1038/srep28468)
Supplement: Supplementary Information [file srep28468-s1.pdf]

<u>Supplementary Information</u>

Spatially resolved photoresponse on individual ZnO nanorods: correlating morphology, defects and conductivity.

K. Bandopadhyay and J. Mitra

S1: Transmission Electron Microscopy

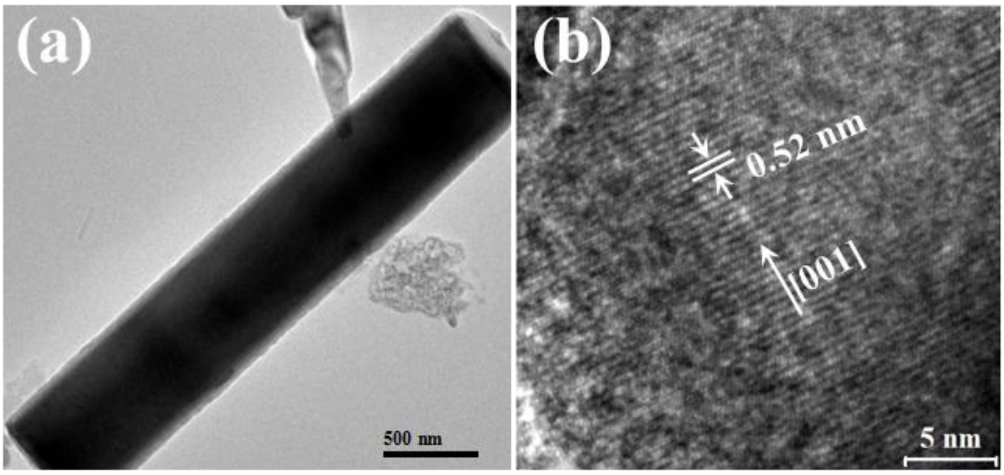

Figure S1: (a) Low resolution and (b) high resolution TEM images of ZnO nanorods.

S2: Grain Size Analysis

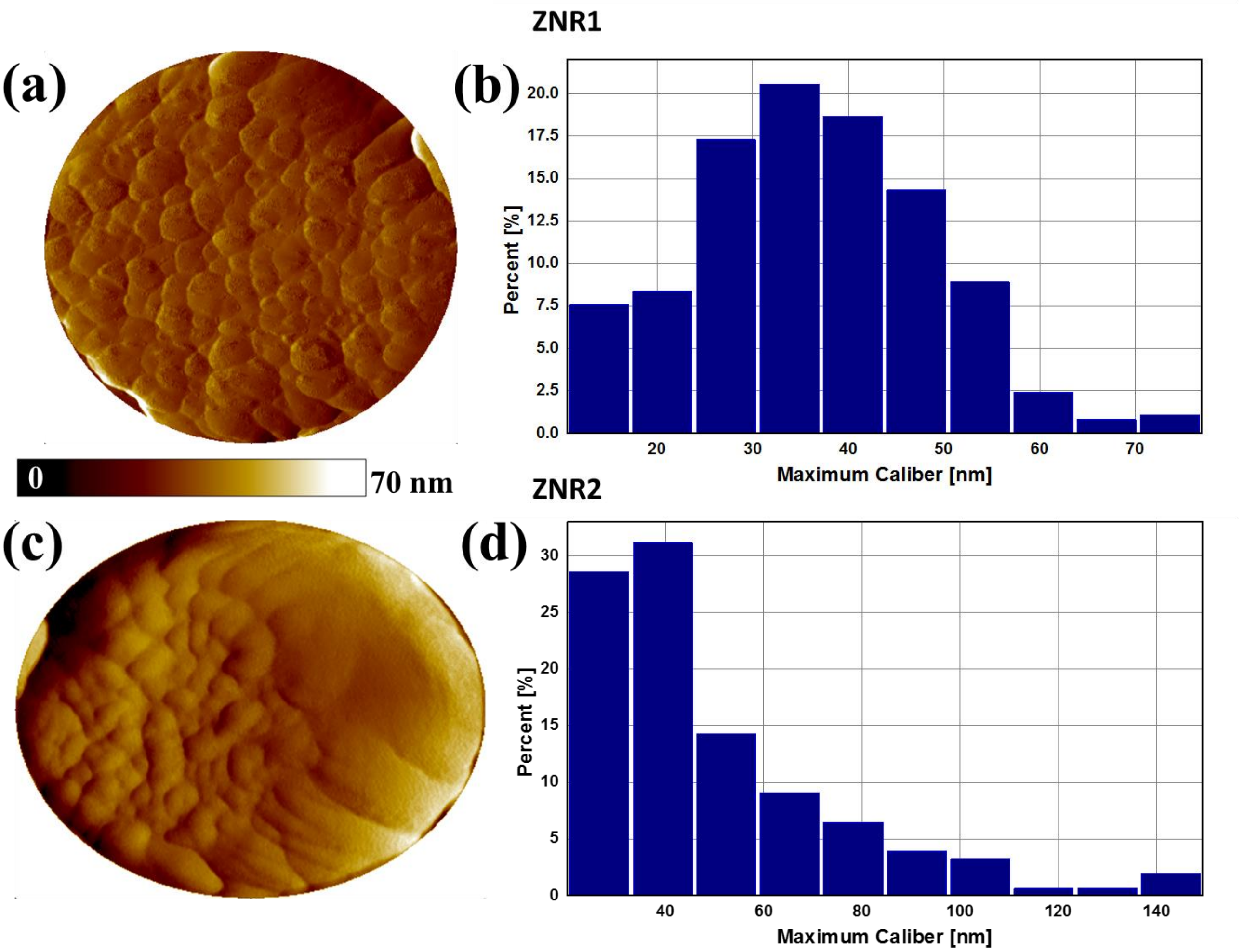

Figure S2: Particle size analysis on top of two different ZnO nanorods, ZNR1 and ZNR2. Surface topography of (a) ZNR1 and (c) ZNR2, showing the granular structure on the top facets of ZnO nanorods. Corresponding percentage distribution of maximum caliber of (b) ZNR1 and (d) ZNR2.

S3: Current Maps

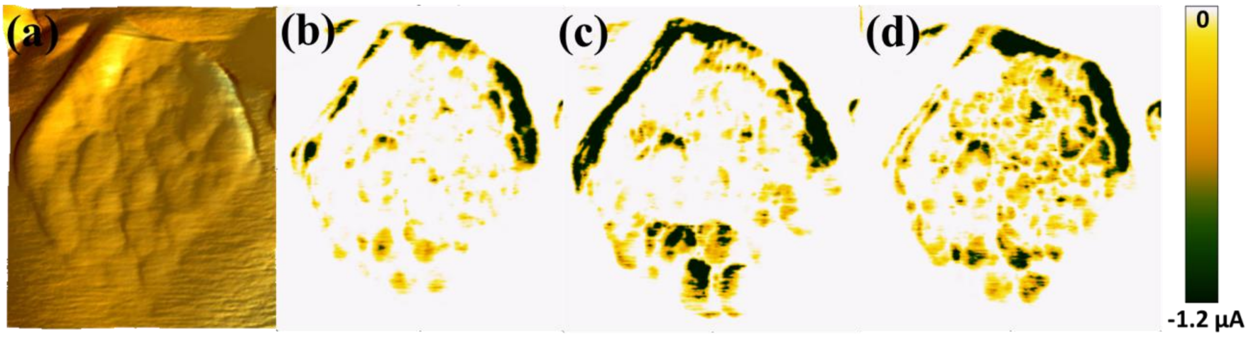

Figure S3: 350 nm ╳ 400 nm (a) 3D rendition of topography of a nanorod (b-d) Current maps acquired in (b) the dark, (c) with 532 nm and (d) with 355 nm excitation. Scan size 366 nm × 415 nm, sample bias – 2 V.

Note: compare current

S4: Conductance (dI/dV) Maps

The conductance or dI/dV maps (CMAPs) i.e. 2D plots of local dI/dV, at a fixed dc sample bias (V_{DC}), were performed using a lock-in amplifier to detect a voltage proportional to the local dI/dV. In this technique the dc sample bias (V_{DC}) is modulated with a sinusoidal ac bias (V_{AC}), such that $(V_{AC})_{rms} \leq$ 5% of V_{DC}. A digital signal generator has been used to source the $V_{AC} = v_0 \sin\omega_0 t$, such that the total sample bias is given by;

$$V(t) = V_{DC} + v_0 sin\omega t \tag{S1}$$

In the present study we have typically used V_{DC} = - 2 V and $(V_{AC})_{rms}$ = 100 mV. For other values of V_{DC} the ac signal has been adjusted suitably. The modulated bias generates a sinusoidal response in the current, which may be expanded into a Taylor series as;

$$I(t) = I_{V_{DC}} + \left(\frac{dI}{dV}\right)_{V_{DC}} v_0 sin\omega t + \frac{1}{2!}\left(\frac{d^2I}{dV^2}\right)_{V_{DC}} v_0^2 sin^2\omega t + \cdots \tag{S2}$$

For small values of modulation i.e. $(V_{AC})_{rms} \leq$ 5% of V_{DC}, the 2nd and higher order terms may neglected with the 1st order term providing a sinusoidal current signal at the input modulation frequency ($f = \omega/2\pi$) as given in equation S1. The current signal is amplified and converted to an equivalent voltage by the current amplifier with a set gain. The voltage output of the current amplifier is then fed to a digital lock-in amplifier that yields the V_{rms} of the in-phase voltage, locked at the input frequency (f). The V_{rms} is proportional to the local *dI/dV* at V_{DC} and is recorded simultaneously with the topography map. The recorded data is then plotted as a function of position to generate the CMAPs. Note that the time constant of the lock-in was always kept at ≥ 3/*f*.

The CMAP is thus a plot of a voltage proportional to the local d*I*/d*V* at the fixed V_{DC}. Evidently the contrast in the CMAP generated shows the 2D variation in the local junction conductance, which is decided by the local electron density and the charge transfer probability. CMAP measurements are more commonly used in the context of scanning tunneling spectroscopy, where the tunnel conductance is directly proportional to the local electron density of states. The CMAP data presented here may be analogously interpreted in the context of the CAFM, especially in the tunneling regime. Note, the *IV* characteristics (figure 3) shows a crossover from thermionic transport to tunneling transport for sample bias |*V*| > 2 V.

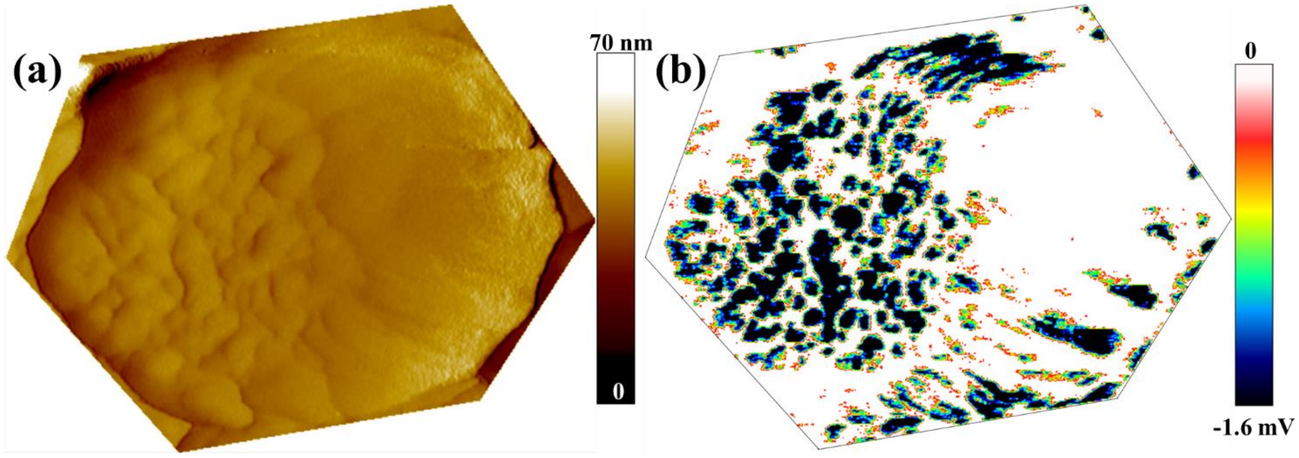

Figure S4: (a) Topography map of a single ZnO nanorod (b) Photoconductance (≡ Voltage) map under λ_1 (355 nm) illumination, which shows that the high photoconductivity is dominantly localized at the centre of the individual photoactive grains than at the grain edges.

Supplementary Movie_M1: In the attached movie the image alternates between the recorded topography and conductance (*dI/dV*) map shown in figure S4, evidencing the correlation between granularity and photoactivity localization.

S5: CMAPs under illumination:

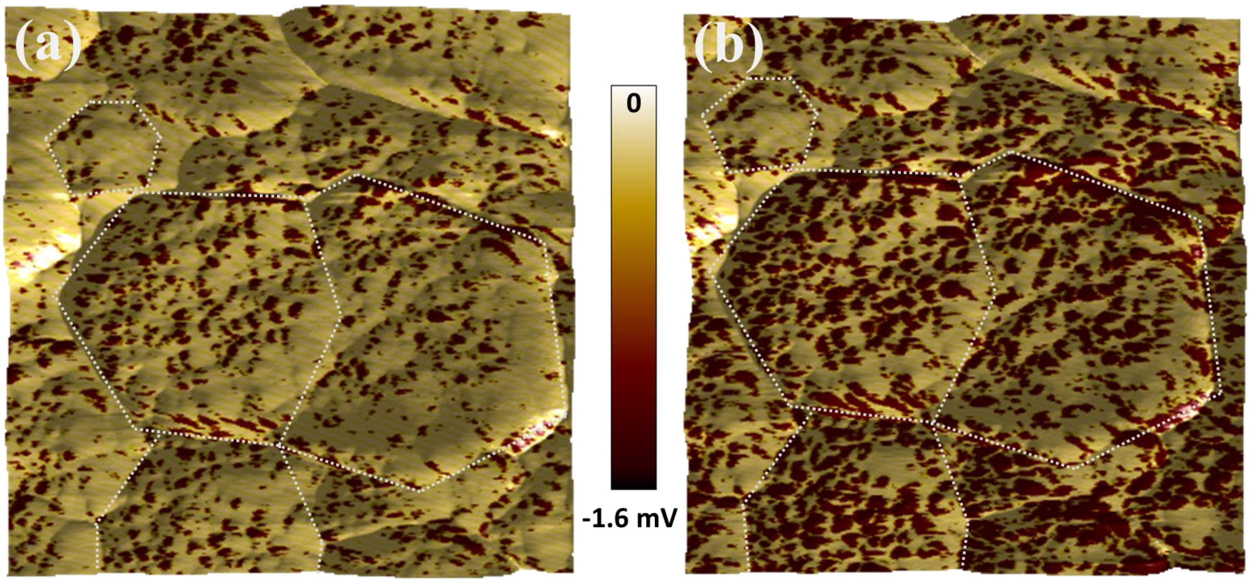

Figure S5: Atomic force microscopy, 1μm × 1μm topography (overlaid with CMAP) showing the hexagonal top surface of multiple ZnO nanorods of different sizes varying from 200 nm to 450 nm. The white dotted lines demarcate the edge of some nanorods for clarity in shape and size. The corresponding CMAPs were obtained in the (a) dark and with (b) 355 nm excitation.

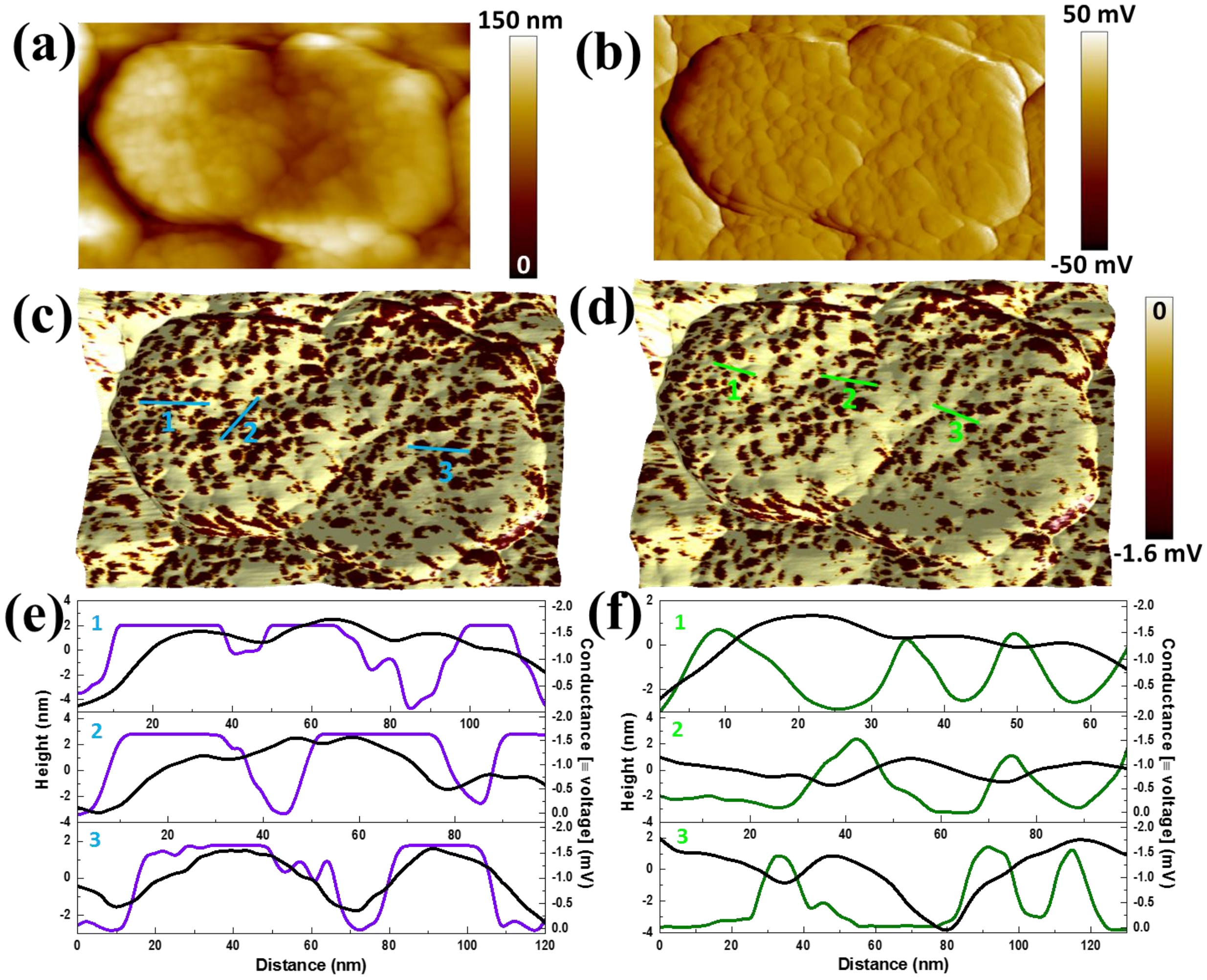

Figure S6: Atomic force microscopy images (a) topography (b) deflection error. 3D topography overlaid with CMAP acquired (c) with 355 nm excitation and (d) with 532 nm excitation. (e-f) simultaneously generated topography and CMAP line-scans from (c) and (d) respectively, showing the correlation of photoresponse (e) with grain centres for 355 nm excitation and (f) with grain edges for 532 nm excitation. Scan size 1µm 600 nm. CMAPs taken with - 2 V sample bias.
